# Sepia

**Published:** 1894-09

**Authors:** 

**Affiliations:** Hamburg


### SEPIA.

Dr. Hesse, Hamburg.

[Translated by A. McNeil, M. D., San Francisco. From *Die Allg. Hom. Zeitung.*]

Bœnninghausen says (1860) that "Sepia occupies a prominent place among our polychrests." Hahnemann calls it "One of the chief anti-psorics." Farrington, "a remedy of invaluable worth." Carroll Dunham says, "Sepia is one of our most important remedies." It was interesting to learn from Dunham's *Lectures on Materia Medica* that Hippocrates highly valued Sepia as a remedy for dysmenorrhœa and diseases of woman in general. Galen recommended Sepia as a tonic and stomachic; Marcellus for kidney gravel, which Dunham calls a remarkable prediction resting on a deduction on the basis of our law of the similars.

Kunkel describes the Sepia constitution in his terse, appropriate style: "Individuals *with dark hair and skin, with a strong disposition to sweat*, particularly *on the back, in the axillæ*, between *the mammæ and on the genitals; pale, yellow face with dirty-looking yellow brown spots around the mouth and on the forehead, swift flashes of heat; disposition to neuralgia; headache* particularly *in the morning* on awakening, which frequently passes off after rising, and often attended by *nausea* and *vomit-*

*ing* and on *awakening heaviness of the head*, and is not *refreshed by sleep.*"

The headache only seldom appears daily, usually every eight to fourteen days. During the attacks the patient wishes to lie down and be quiet, at other times *physical restlessness* which compels them to *rise from the chair and walk about.* They *complain* of *stiffness* on *rising from* the *chair*, so that it is difficult to walk.

Warm air both out-of-doors and in a warm room is intolerable. Foggy weather, north and east (moist in Europe) wind; acid and fatty foods disagree.

*In the three days before menses pelvic pains and aggravation of all symptoms.*

Kunkel agrees with Bœnninghausen in saying that persons with dark hair are particularly susceptible to the beneficial action of Sepia. However it acts if indicated also in blondes (Bœnninghausen puts Sepia in the highest rank for dark persons and the lowest for fair.—*Trans.*), the fat as well as the emaciated, men as well as women, and the aged as well as children.

We often observe the characteristic restlessness of patients in our offices: The children cannot remain quiet for a minute, even adults, in spite of their self-control, find it difficult to sit quietly in their chairs. It is a necessity for them to move about.

Their walk is often hasty, more like a run than a walk. This is a characteristic quality of nearly all Sepia complaints. They are ameliorated by movement, by walking, running, dancing, turning, and gymnastic exercises. Sepia in this regard of amelioration by *rapid* movement, running, dancing, violent motion, stands almost alone. Pulsatilla has improvement by slow motion and also Ferrum.

The Rhus patient cannot endure rest. He must move, but he cannot bear the severe exertion that characterizes Sepia. The faster the Sepia patient runs and the longer the better he feels. In the beginning of the exertions many complaints, as palpitation of the heart, affection of the stomach, difficult respiration, and sciatica are aggravated, which pass away when the exercise is continued, but return all the worse in the rest which follows.

Lying is proportionally tolerable. Sitting, particularly sitting bent, is the worst in gastric and respiratory complaints.

I cured with Sepia dyspnœa and palpitation relieved by running and dancing; gastric complaints disappearing when running and dancing, and coryza, which passed away after dancing a long time.

Sitting long and the talking of others is disagreeable, long dinners are intolerable, attendance on concerts and theatres often impossible on account of the long sitting still, the bad air, and presence of many people.

If either children or adults are compelled to sit still they have the irresistible impulse to move their legs and pull their hair, or play with some object in their hands. It relieves them when a part of the body is in motion. This restlessness borders on the pathological restlessness of St. Vitus's dance, and in fact Sepia has done good service in this disease, either in alternation (in the Hahnemanian sense.—*Trans.*) with Stramonium or following it to remove the remainder of the disease.

In the mercantile world of this city, where the difficult struggle for existence in the modern high pressure of this commercial metropolis keeps body and mind in a constant strain, I find Sepia frequently indicated for the new disease neurasthenia. Such a patient sleeps with difficulty and without being refreshed thereby, eats rapidly, walks fast, and works hard. In the whirl of business he does not feel any distress, but when at rest it haunts him. Emotions affect him greatly. Inactivity torments him, and Sunday is to him the hardest of all days.

If I treated exhaustively the sphere of action of Sepia I would have to travel through the entire field of pathology. It is in the arena of chronic diseases one of the most powerful of our weapons.

---

BEARING down in the region of the uterus as if everything would come out. Must cross the legs to prevent it. Sense of weight in the anus. Weak gone feeling in the stomach. Brick-dust sediment in the urine. Sepia.—*Guernsey's Keynotes.*

